# Ensemble of Bayesian alphabets via constraint weight optimization strategy improves genomic prediction accuracy

**DOI:** 10.1093/g3journal/jkaf150

**Published:** 2025-07-29

**Authors:** Prabina Kumar Meher, Upendra Kumar Pradhan, Mrinmoy Ray, Ajit Gupta, Rajender Parsad, Pushpendra Kumar Gupta

**Affiliations:** Division of Statistical Genetics, ICAR—Indian Agricultural Statistics Research Institute, PUSA, New Delhi 110012, India; Division of Statistical Genetics, ICAR—Indian Agricultural Statistics Research Institute, PUSA, New Delhi 110012, India; Division of Forecasting and Agricultural Systems Modeling, ICAR—Indian Agricultural Statistics Research Institute, PUSA, New Delhi 110012, India; Division of Statistical Genetics, ICAR—Indian Agricultural Statistics Research Institute, PUSA, New Delhi 110012, India; ICAR—Indian Agricultural Statistics Research Institute, PUSA, New Delhi 110012, India; Department of Genetics and Plant Breeding, Chaudhary Charan Singh University, Meerut 250004, India

**Keywords:** genomic prediction, ensemble model, meta-learning, Bayesian model, genetic algorithm

## Abstract

This study proposes a weight optimization-based ensemble framework aimed at improving genomic prediction accuracy. It incorporates 8 Bayesian models—BayesA, BayesB, BayesC, BayesBpi, BayesCpi, BayesR, BayesL, and BayesRR in the ensemble framework, where the weight assigned to each model was optimized using genetic algorithm method. The performance of the ensemble model, named EnBayes, was evaluated on 18 datasets from 4 crop species, showing improved prediction accuracy compared to individual Bayesian models. New objective functions were proposed to improve prediction accuracy in terms of both Pearson's correlation coefficient and mean square error. The accuracy of the ensemble model was found to be associated with the number of models considered in the framework, where a few more accurate models achieved similar accuracy as that of more number of less accurate models. Additionally, over-bias and under-bias models also influenced the biasness of the ensemble model's accuracy. The study also explored a meta-learning approach using Bayesian models as base learners and random forest, quantile regression forest, and ridge regression as meta-learners, with the EnBayes model outperforming this approach. While traditional genomic prediction models GBLUP and rrBLUP and machine learning models support vector machine, random forest, extreme gradient boosting, and light gradient boosting were included in the ensemble framework in addition to Bayesian models, the ensemble model achieved higher accuracy as compared to the individual Bayesian, BLUP, and machine learning models. We believe that EnBayes would contribute significantly to ongoing efforts on improving genomic prediction accuracy.

## Introduction

Traditional crop improvement programs involve hybridization followed by growing segregating populations, where selection of superior plants is guided by observable desirable traits. However, in recent years, the availability of molecular markers in large number, particularly the single nucleotide polymorphisms (SNPs) based on DNA-sequencing technology, provides valuable resource for genotype selection (Crossa et al. 2017). This technological progress has allowed breeders to evaluate plant performance based on DNA-based markers profiles rather than just observable traits. The development of markers to be used for marker assisted selection (MAS) initially involved 2 important approaches, the first including linkage-based interval mapping of quantitative trait loci (QTLs), where 1 or more DNA markers linked to QTLs are selected, and the second including linkage-disequilibrium (LD) based genome-wide association studies. In both cases, markers closely associated with QTLs for desired traits are utilized (Zhu et al. 2008; Tibbs Cortes et al. 2021). However, the application of MAS is based on a small number of major QTLs, leaving many small-effect QTLs in complex traits unutilized (Jannink et al. 2010). In other words, MAS has limitations, particularly when dealing with complex traits, which are influenced by many genes including few major genes and a large number of minor genes (Bernardo 2021), thus reducing the accuracy of selection.

More recently, genomic selection (GS; Meuwissen et al. 2001) has become a powerful tool in plant breeding, particularly with the development of genome-wide SNPs. Unlike MAS, which focuses on a limited number of markers linked to major QTLs, GS employs a large number of genome-wide SNPs to assess the overall genetic merit of individual plants, capturing the effects of most QTLs (both major and minor) associated with the trait of interest (Bernardo and Yu 2007; Heffner et al. 2009). GS models aim to capture a significant proportion of heritable phenotypic variation using genome-wide marker data to assign genomic estimated breeding values (GEBVs) to available individuals (Meuwissen et al. 2001; Jannink et al. 2010). Unlike QTL mapping, which primarily identifies only large additive genetic effects, GS models incorporate major genetic effects of genome-wide significance. This approach allows genome-wide markers to account for a large proportion of heritable variation, particularly in traits with complex genetic architectures (Goddard et al. 2016). GS has the potential to reduce the cost per breeding cycle, increase selection intensity and accuracy, and significantly shorten the time required to develop a new cultivar compared to phenotypic-based selection (Crossa et al. 2017; Edwards et al. 2019). GS tools are expected to drive major improvements in plant breeding in the next few decades (Mackay et al. 2021).

The accuracy of GS depends upon the accuracy of genomic prediction (GP), which is estimated using a data-driven technique and has gained widespread acceptance for accelerating genetic gains in plant breeding programs (Desta and Ortiz 2014; Bassi et al. 2016; Xu et al. 2020). GP leverages advanced statistical and machine learning models to estimate breeding values from genome-wide markers and select individuals in a segregating population in a breeding program. The GP is worked out through a regression approach, where the relationship between genotypic and observed phenotypic values is modeled (Jannink et al. 2010) and predict breeding values for traits in a target population that only has genotypic information, without the need of recording phenotypic data.

The performance of GP models is primarily assessed using Pearson's correlation coefficient (PCC) and mean squared error (MSE); a higher PCC and lower MSE indicate a better model. The core of GP is the statistical model, where the goodness of the model greatly affects the accuracy and efficiency (Banerjee et al. 2020). Although, GP accuracy has steadily improved through ongoing optimizations of statistical models, 2 significant challenges remain: computational accuracy and efficiency (Yin et al. 2019). Therefore, the exploration of more robust GP models is a well-identified research area.

Meuwissen et al. (2001), while proposing GS, initially introduced a mixed model for best linear unbiased prediction (BLUP), along with 2 Bayesian models (BayesA and BayesB). The 2 Bayesian models have evolved into what is now known as the Bayesian alphabet (Gianola et al. 2009; Gianola 2013). Each model is based on different assumptions about the genetic architecture of underlying trait variability. The versatility offered by different formulations of an additive model within the Bayesian alphabet framework has made them a popular choice for predicting complex traits in both livestock (El Jabri et al. 2019; Wang et al. 2019; van den Berg et al. 2020) and crops (Kwong et al. 2017; Shikha et al. 2017; Deomano et al. 2020). Nonetheless, each Bayesian model has its own strength and weakness depending upon the genetic architecture of the underlying trait. In other words, there is not a “one-size-fits-all” approach for GP across different traits and species. Therefore, it is necessary to apply the available GP models and select the one that offers the highest prediction accuracy for the trait of interest. However, accuracy of GP can be enhanced by combining the outputs of all the models in an ensemble framework. In existing studies, 2 types of ensemble strategies have been mostly employed. In the first category, the output is the weighted average of the accuracies of individual models, where the weights assigned to different models are either determined through constraint weight optimization strategy or based on certain rule. For instance, Kick and Washburn (2023) employed uniform weights and weights inversely proportional to standard deviation, variance, and root mean square error of the training dataset, to combine outputs from BLUP, machine learning (ML), and deep learning models. In another study, Gu et al. (2024) used genomic BLUP (GBLUP), BayesA, BayesB, and BayesCpi as base models and employed a hybrid of differential evolution and particle swarm optimization (PSO) to determine the optimal weights for averaging the predictions of the base models. In the second category of ensemble, meta-learning approach has been adopted to combine the output of several base learners in order to improve the GP accuracy (Liang et al. 2021; Yu et al. 2021; Nascimento et al. 2024).

Based on the studies mentioned above, it is clear that ensemble learning models outperform individual base learners in GP. Existing ensemble GP models have been employed either for the maximization of the PCC or minimization of MSE. None of these studies have considered maximization of PCC and minimization of MSE at the same time. Further, the ML algorithms have been mostly preferred as the base learners in existing ensemble studies, despite computationally demanding when dealing with high-dimensional marker data. On the other hand, there are several variants of Bayesian models available for GP and only a few (typically 2 or 3) of them have been used within an ensemble framework. Also, no comparative analysis has been performed to analyze whether constraint weight optimization strategy or meta-learning approach is better to improve the GP accuracy in an ensemble framework. In this study, we have developed an ensemble framework aimed at improving GP accuracy by incorporating 8 different Bayesian models. New objective functions were proposed to determine the weights which could simultaneously improve the GP accuracy in terms of both PCC and MSE. The genetic algorithm (GA; Katoch et al. 2021) optimization technique was implemented to determine the optimum weight for each Bayesian model. The proposed ensemble approach was also compared with existing traditional models GBLUP, ridge regression BLUP (rrBLUP), reproducing kernel Hilbert space (RKHS) and ML models such as support vector machine (SVM), extreme gradient boosting (XGB), and random forest (RF). We also compared the optimized GP accuracy obtained through constraint weight optimization technique and meta-learning approach. The developed ensemble approach was further evaluated by considering rrBLUP, GBLUP, SVM, RF, XGB, and light gradient boosting (LGB) models in the ensemble framework, in addition to the 8 Bayesian models. The present study is believed to supplement the existing studies for GP with improved accuracy.

## Materials and methods

### Ensemble genomic prediction strategy

Let y be the observed value of the given phenotypic trait, and $\hat{y_{j}}$ be the corresponding predicted phenotypic value by the jth genomic prediction model (j=1,2,…,k). The proposed ensemble approach considers assigning weight (*w*) to the predicted phenotypic values in such a way that GP accuracy is maximized. The PCC and MSE are the 2 important metrics that have been widely used to assess the GP accuracy in existing studies. In other words, higher accuracy means higher PCC and lower MSE. Thus, the proposed ensemble approach was formulated as a constraint optimization problem to maximize the PCC and minimize the MSE. In other words, the optimization problem involves maximization of the objective function $f_{1}(w)$ and minimization of $f_{2}(w)$, where


$$f_{1}(w)=\frac{\mathrm{cov}\left(y,\;\;\;\sum_{\;j=1}^{k}\;w_{j}\hat{y_{j}}\right)}{\sqrt{\mathrm{var}(y)}\;\sqrt{\mathrm{var}\left(\sum_{\;j=1}^{k}\;w_{j}\hat{y_{j}}\right)}};\;j\;=1,2,\ldots ,k\;\mathrm{and}$$

$$f_{2}(w)=\frac{1}{n}\sum_{i=1}^{n}{\left(y_{i}-\sum_{\;j=1}^{k}w_{j}\hat{y_{ij}}\right)}^{2};\;i=1,\;2,\;\ldots ,\;n\;(\mathrm{number}\;\mathrm{of}\;\mathrm{lines}).$$

However, the maximization of PCC may not always ensure that MSE is low and minimization of MSE may not always ensure that PCC is high. Therefore, achieving higher GP accuracy in terms of both the metrics is challenging. To address this issue, we considered maximization of 2 more objective functions $f_{3}(w)$ and $f_{4}(w)$, where $f_{3}(w)=f_{1}(w)-f_{2}(w)$ and $f_{4}(w)=\frac{\;f_{1}(w)}{\;f_{2}(w)}$. Here, weights are subjected to the constraints that $0\leq w_{1},w_{2},\ldots ,w_{k}\leq 1$ and $\sum_{\;j=1}^{k}w_{j}=1.$ Optimized values of the weights were determined using the GA optimization technique (Katoch et al. 2021). The steps involved in GA technique are provided in Box 1. We utilized the R-package *GA* (Scrucca 2013) to implement the GA. As far as parameter setting is concerned, we considered the size of initial population m = 300, crossover rate = 0.2, mutation rate = 0.1, and maximum number of iteration = 1,000. In the ensemble framework, we considered 8 Bayesian GP models which include BayesA (Meuwissen et al. 2001), BayesB (Meuwissen et al. 2001), BayesC (Habier et al. 2011), BayesBpi (Meuwissen et al. 2001), BayesCpi (Meuwissen et al. 2001), BayesR (Moser et al. 2015), BayesL (Yi and Xu 2008), and BayesRR (de Los Campos et al. 2013).

Box 1. **Steps involved for determination of weight using genetic algorithm (GA).**

1. Initialization of parameters
  - Initialize a population of *m* individuals (chromosomes), where each individual is a *k*-dimensional vector of weights ($w_{j};j=1,2,\ldots ,m$) corresponding to *k* GP models
  - Randomly initialize the weights for each solution/individual within the range [0,1]
2. Evaluation of fitness/objective function
  - Determine the value of the fitness function {$f_{1}(w),\;f_{2}(w),\;f_{3}(w),\;f_{4}(w)$} for each weight vector (individual/chromosome) in the population
3. Selection, crossover, mutation, and updation
  - Select the pair of individuals based on the higher values of the fitness function
  - Define crossover rate $p_{c}$ to produce offsprings from the selected pair of individuals. For instance, let $C_{1}:[w_{11},w_{12},w_{13}]$ and $C_{2}:[w_{21},w_{22},w_{23}]$ be the 2 selected chromosomes for 3 GP models. Then, using the single point crossover after the first gene, the offspring will be $OS_{1}:[w_{11},w_{22},w_{13}]$ and $OS_{2}:[w_{21},w_{12},w_{23}]$
  - Define mutation rate and randomly alter the genes in the offspring. For example, in the first offspring ($OS_{1}$), the second gene is mutated which means change in the $w_{22}$ value by a small random value within range [0,1]. In other words, the new first offspring will be $OS_{1n}:[w_{11},w_{22}+0.001,w_{13}]$
  - Retain the best individual (weight vector) from the old population and update the rest with the new offspring (weight vector)
  - Continue updating the fitness function followed by selection, mutation, and crossover until a stopping criterion is reached i.e. a maximum number of iterations or maximum fitness level

**Fig. 1.**
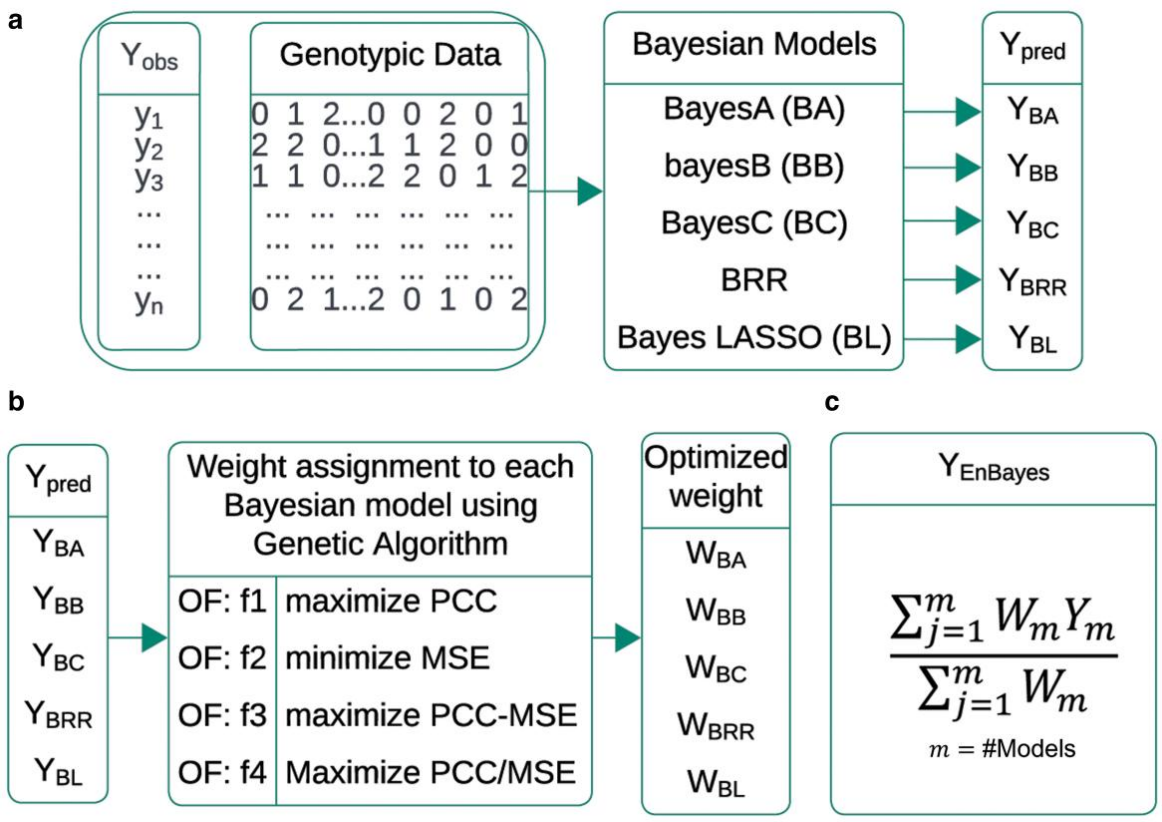
Workflow of the proposed ensemble approach. a) Prediction of trait of interest using different Bayesian models. b) Determination of weight assigned to the predicted trait values by different Bayesian models. c) Prediction of trait values using ensemble model.

For the linear mixed effect model y=μ1+Xβ+Zu+ε, where y is the vector of phenotypic values, X is the design matrix for the fixed effects, *β* is the vector of fixed effects, Z is the design matrix for the random effects (genetic markers/SNPs), u is the vector of random effects, and ε is the residual errors, the Bayesian approach deals with estimating posterior distribution of the marker effects ($u_{j}$) given the data i.e. $\;p(u_{j}|y)\propto p(y|u_{j}).p(u_{j}|\theta )$, where $p(y|u_{j})$ is the likelihood of the observed phenotypes (*y*) given the marker effects ($u_{j}$) and $p(u_{j}|\theta )$ is the prior distribution of the marker effects specified by the model. The 8 Bayesian models mainly differ in assuming prior distribution of the marker effects and variance of the markers effect size. The assumptions for the prior distribution of the marker effects and their variances are listed in Table 1. Except BayesL, the variance of the marker effects is assumed to follow inverse chi-square distribution i.e. $\sigma^{2}∼\chi^{-2}(\nu ,S)$ or $\sigma_{j}^{2}∼\chi^{-2}(\nu ,S)$, where *ν* is the degree of freedom with smaller value representing a larger marker effect and *S* is the scale parameter with larger value indicates a higher probability of larger variance values and smaller value indicates smaller variance values. In case of BayesL, $\sigma_{j}^{2}∼\mathrm{Exp}(\lambda^{2}/2)$. The estimated proportion of marker effects $\hat{\pi }$ are assumed to follow Beta distribution i.e. $\hat{\pi }∼\mathrm{Beta}(\eta_{1},\eta_{2})$, where $\eta_{1}$ and $\eta_{2}$ are the shape parameters with $\eta_{1}>\eta_{2}$ denoting skewed distribution toward 1 indicating a prior belief that markers are more likely to have nonzero effects. If $\eta_{1}<\eta_{2}$, the distribution is skewed toward 0, indicating a prior belief that most of the markers have zero effects. If the marker effects are assumed to be mixture of more than 2 normal distribution, then $\hat{\pi }∼\mathrm{Dirichlet}(\eta )$, where *η* is a positive integer. The residual effect is also assumed to follow normal distribution i.e. $\epsilon ∼N(0,\sigma_{\epsilon }^{2})$ and $\sigma_{\epsilon }^{2}∼\chi^{-2}(\nu_{\epsilon },S_{\epsilon })$. The *hibayes* R-package (version 1.0.0 available at https://cran.r-project.org/src/contrib/Archive/hibayes/) (Yin et al. 2022) was used to implement the considered 8 Bayesian models. The MCMC algorithm with nburn = 14,000, niter = 20,000, and thin = 100 was utilized for implementation of Bayesian models for GP. A graphical representation of the proposed ensemble approach is shown in Fig. 1. All the source code are available at https://github.com/PrabinaMeher/EnBayes for reproducibility of the developed approach.

**Table 1.** List of the Bayesian model with their assumption regarding the prior distribution of the marker effects.

Method	Prior distribution of marker effects
BayesA	All the makers have nonzero effect and unequal variance for all the markers effect $u_{j}∼N(0,\sigma_{j}^{2})$
BayesB	Most of the markers (95%) have zero effect and only a small proportion of markers (5%) have nonzero effect, and unequal variances for all the nonzero marker effects $u_{j}∼\pi_{0}.N(0,\sigma_{j}^{2})+(1-\pi_{0}).\delta_{0}$ $\pi_{0}=0.05$, $\delta_{0}$ indicates a point mass at zero, indicating no effect
BayesC	Most of the markers (95%) have zero effect and only a small proportion of markers (5%) have nonzero effect, and equal variances for all the marker effects $u_{j}∼\pi_{0}.N(0,\sigma^{2})+(1-\pi_{0}).\delta_{0}$ $\pi_{0}=0.05$, $\delta_{0}$ indicates a point mass at zero, indicating no effect
BayesBpi	Most of the markers (95%) have zero effect and only a small proportion of markers (5%) have nonzero effect, and unequal variances for all the marker effects $u_{j}∼\pi .N\left(0,\sigma_{j}^{2}\right)+\left(1-\pi \right).\delta_{0}$ Here, $\hat{\pi }$ is not fixed and is estimated through iterative process, $\delta_{0}$ indicates a point mass at zero, indicating no effect
BayesCpi	Most of the markers (95%) have zero effect and only a small proportion of markers (5%) have nonzero effect, and equal variances for all the marker effects $u_{j}∼\pi .N\left(0,\sigma^{2}\right)+\left(1-\pi \right).\delta_{0}$ $\hat{\pi }$ is not fixed and is estimated through iterative process, $\delta_{0}$ indicates a point mass at zero, indicating no effect
BayesR	Most of the markers (95%) have zero effect and only a small proportion of markers (5%) have nonzero effect. The prior distribution of the nonzero markers effects is assumed to follow mixture normal distribution with equal variance for the nonzero marker effects $u_{j}∼\pi_{1}.N(0,10^{-3}\sigma^{2})+\pi_{2}.N(0,10^{-2}\sigma^{2})+\pi_{3}.N(0,10^{-1}\sigma^{2})+(1-\pi_{1}-\pi_{2}-\pi_{3}).\delta_{0}$ $\pi_{1}=0.0001,\pi_{2}=0,001,\pi_{3}=0.01$ and $\delta_{0}$ indicates a point mass at zero, indicating no effect
BayesRR	All the makers have nonzero effect and equal variance for all the markers effect $u_{j}∼N(0,\sigma^{2})$
BayesL	All the markers have nonzero effect with larger number of markers have small effect and some markers have larger effects. Different variances for different markers effect size $u_{j}∼N(0,\sigma_{j}^{2})$

All the Bayesian models were implemented with parameters nburn = 14,000, niter = 20,000, and thin = 100.

### Phenotypic and genotypic datasets

We evaluated the proposed ensemble approach on 18 different datasets from 4 different crops including rice, wheat, maize, and groundnut. These datasets include both genotypic and phenotypic information, sourced from previously published studies. The genotypic data encompassed diverse marker types such as SNPs, Genotyping-by-Sequencing (GBS), and Diversity Arrays Technology (DArT) markers. A brief description of each dataset is provided below:

Wheat yield dataset: The genotypic dataset comprises of 599 wheat lines, each genotyped using 1,447 DArT markers. A total of 1,279 markers obtained after excluding markers with <5% minor allele frequency (MAF) were utilized in this study. Phenotypic dataset comprises grain yield trait recorded in 4 mega-environments, denoted as WY1, WY2, WY3, and WY4. This dataset was obtained from Crossa et al. (2010) and is readily available through the R-package *BGLR* (Pérez and de los Campos 2014).
Maize yield dataset: Sourced from Crossa et al. (2010), the phenotypic dataset includes grain yield under well-watered (M_WW) and severe-stress (M_SS) conditions for 264 tropical maize lines from the Drought Tolerance Maize for Africa project under CIMMYT's Global Maize Program. Genotypic data comprises 1,148 SNP markers, obtained after removing those with <5% MAF and imputing missing data. The phenotypic and genotypic datasets are available at http://www.genetics.org/cgi/content/full/genetics.110.118521/DC1.
Rice yield dataset: The phenotypic data comprising grain yield for 327 *Indica* rice genotypes for the years 2011 and 2012 were obtained from the study of Monteverde et al. (2019), respectively, represented as RY11 and RY12. Though 92,430 GBS markers are available in the original dataset after imputation and excluding markers having MAF ≤ 0.05, only the first 16,383 markers were considered in this study to avoid computational complexity. Both phenotypic and genotypic datasets are publicly available at CIMMYT data repository.
Groundnut yield dataset: The phenotypic and genotypic datasets for 318 groundnut genotypes evaluated across 4 environments (Aliyarnagar-Rainy, Jalgaon-Rainy, ICRISAT-Rainy, and ICRISAT-Post Rainy) during 2015 were obtained from the study of Pandey et al. (2020). The phenotypic dataset considered in this study includes yield per hectare denoted as GY1, GY2, GY3, and GY4 for the 4 environments, respectively. As far as genotypic dataset is concerned, 8,268 SNP markers, coded as 0, 1, or 2, are available following quality control. The dataset is accessible at CIMMYT data repository.
Wheat micro-nutrient dataset: Genotypic and phenotypic datasets for 330 spring wheat lines were retrieved from Velu et al. (2016). Phenotypic traits include grain iron (Fe) and zinc (Zn) concentrations, evaluated across 3 Indian wheat-growing regions: Punjab Agricultural University (PAU), Ludhiana; Indian Institute of Wheat and Barley Research (IIWBR), Karnal; and Banaras Hindu University (BHU), Varanasi during 2012. These traits are denoted as PAU_Fe, PAU_Zn, IIWBR_Fe, IIWBR_Zn, BHU_Fe, and BHU_Zn. SNP markers were coded as 0, 1, and 0.5 for homozygote reference, homozygote alternate, and heterozygote, respectively. Markers with <5% MAF were excluded, leaving 20,595 markers after imputation for 324 genotypes. This dataset was obtained by email request to Dr. Govindan Velu (velu@cgiar.org) on August 2, 2023, and is not publicly available.

All the processed genotypic and phenotypic datasets used in this study are made available at https://github.com/PrabinaMeher/EnBayes.

### Cross-validation and performance metrics

The repeated random cross-validation approach was used to evaluate the predictive performance of GP models, as it reduces the risk of over fitting and improves the generalizability of the prediction. In this approach, the dataset was randomly split into training and testing subsets 100 times, with 80% of the data allocated for training and 20% for testing. For each iteration, the model was trained using the training subset and its performance was evaluated on the corresponding test subset. The final predictive accuracy was calculated by averaging the results across all repetitions, providing a more robust and reliable estimate of the model's performance. To measure GP accuracy, PCC was calculated between the observed values (*y* e.g. true genetic values or phenotypes) and the predicted values ( e.g. GEBVs) using the following formula.


$$\mathrm{PCC}=\frac{\sum_{i=1}^{n}(y_{i}-\bar{y})({\hat{y}}_{i}-\bar{{\hat{y}}_{i}})}{\sqrt{\sum_{i=1}^{n}{\left(y_{i}-\bar{y}\right)}^{2}}\sqrt{\sum_{i=1}^{n}{\left({\hat{y}}_{i}-\bar{{\hat{y}}_{i}}\right)}^{2}}}.$$

Additionally, bias was also assessed for each GP model and was quantified by regressing observed values on predicted values i.e. $y=a+b\hat{y}+\epsilon$, where a regression coefficient (*b*) of 1 indicates no bias, b>1 suggest overestimation, and b<1 indicates underestimation.

### Comparison with other GP models

In order to compare the proposed weight optimization strategy (EnBayes) with that of meta-learning approach for ensemble GP, we utilized the Bayesian alphabets as base learners and employed ridge regression (RR; McDonald 2009), quantile regression forest (QRFR; Meinshausen and Ridgeway 2006), and random forest regression (RFR, Breiman 2001) as meta-learners. The RR, QRFR, and RFR have also been used as meta-learner in existing study (Nascimento et al. 2024). Besides, the predictive performance of the developed ensemble approach was also compared against 6 other models including GBLUP (Meuwissen et al. 2001), rrBLUP (Endelman 2011), RKHS (Gianola and van Kaam 2008), SVM (Cortes and Vapnik 1995), RF (Breiman 2001), and XGB (Chen and Guestrin 2016). Among these, GBLUP, rrBLUP, and RKHS represent traditional statistical models, while SVM, RF, and XGB are ML models. In addition to 8 Bayesian models, rrBLUP, GBLUP, SVM, RF, XGB, and light gradient boosting (LGB, Ke et al. 2017) were also included in the ensemble framework to evaluate the performance of ensemble model with diverse GP models in the ensemble framework. The R-package *BGLR* (Pérez de los Campos 2014) was used for implementing GBLUP and RKHS, whereas R-package *rrBLUP* (Endelman 2011) was utilized to implement rrBLUP model. The R-packages *e1071* (Dimitriadou et al. 2008), *xgboost* (Chen 2015), *randomForest* (Liaw and Wiener 2002), and *lightgbm* (Shi et al. 2022) were used for implementing SVM, XGB, RF, and LGB, respectively. The R-packages *quantregForest* (Meinshausen 2017) and *glmnet* (Friedman et al. 2010) were respectively utilized for implementing QRFR and RR models.

## Results

### Accuracy of ensemble model and individual model

**Fig. 2.**
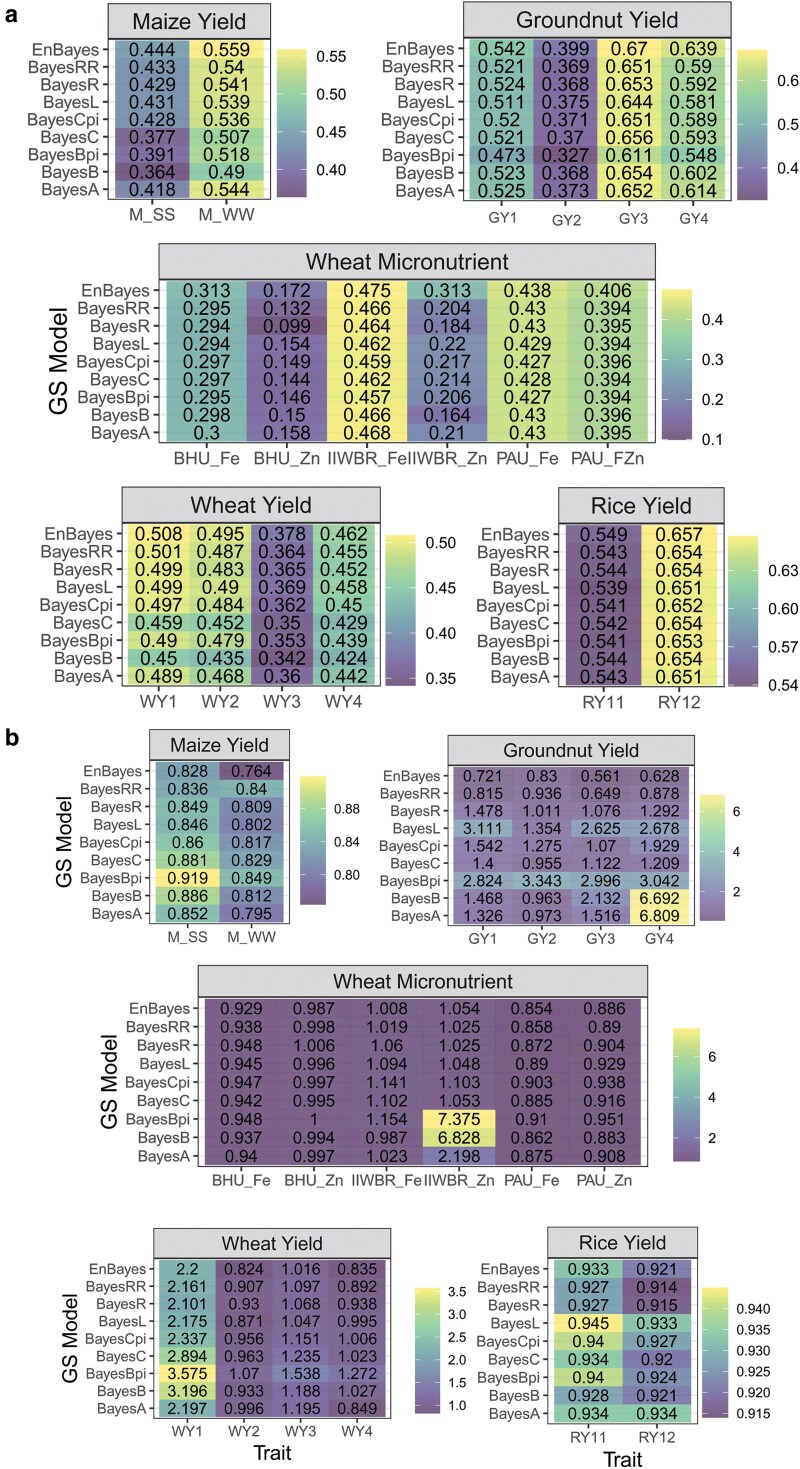
Heat maps of the genomic prediction accuracy of the Bayesian models and the ensemble model (EnBayes) measured in terms of PCC a) and MSE b). The objective functions $f_{1}(w)$ and $f_{2}(w)$ are utilized to measure PCC and MSE, respectively.

For all the 18 traits, the optimized PCC (averaged over 100 replications) of the ensemble model (EnBayes with objective function $f_{1}(w)$) was observed to be higher than that of all the individual Bayesian models (Fig. 2a). Specifically, the EnBayes model achieved higher PCC of 0.444 and 0.559 than that of best performing Bayesian model BayesRR (0.433) and BayesR (0.541) for the traits M_SS and M_WW, respectively (Fig. 2a). In case of nutritional trait, the EnBayes achieved PCC of 0.313, 0.172, 0.475, 0.313, 0.438, and 0.406 against the highest PCC of 0.298, 0.158, 0.468, 0.430, and 0.396 obtained by individual Bayesian model for the traits BHU_Fe, BHU_Zn, IIWBR_Fe, IIWBR_Zn, PAU_Fe, and PAU_Zn, respectively (Fig. 2a). For the wheat yield trait, the highest PCC obtained by Bayesian model was 0.501 (WY1), 0.487 (WY2), 0.369 (WY3), and 0.458 (WY4), whereas the accuracy of the EnBayes was observed to be 0.508, 0.495, 0.378, and 0.462, respectively (Fig. 2a). Similarly for the yield trait of rice, the highest PCC obtained by the individual model was 0.543 (RY11) and 0.654 (RY12) and by the EnBayes model was 0.549 (RY11) and 0.657 (RY12). In terms of MSE (obtained with objective function $f_{2}$(*w*)), the EnBayes model exhibited lower MSE for 13 traits (M_SS, M_WW, GY1, GY2, GY3, GY4, BHU_Fe, BHU_Zn, IIWBR_Fe, PAU_Fe, WY2, WY3, and WY4) compared to the 8 Bayesian models (Fig. 2b). For the remaining 5 traits (IIWBR_Zn, PAU_Zn, WY1, RY11, RY12), the Bayesian models showed lower MSE (Fig. 2b), which may be attributed to possible non-convergence in the MCMC algorithm for some Bayesian models.

### Optimization of genetic algorithm hyper-parameters

For implementing GA, we initially used a population size of 300, crossover rate of 0.2, mutation rate of 0.1, and 1,000 iterations. To further assess the robustness of GP accuracy, measured using PCC obtained with the objective function $f_{1}\;(w)$, we evaluated the GA under varying parameter settings: population sizes of 200, 300, and 500; crossover rates of 0.2, 0.4, and 0.8; mutation rates of 0.05, 0.1, and 0.15; and iterations of 1,000 and 5,000. Results indicated that, except for the higher crossover rate of 0.8, which consistently yielded lower PCC values, the GP accuracies were observed at par across the other parameter combinations (Supplementary Fig. 1). Based on this evaluation, we retained the original GA parameters: population size of 300, crossover rate of 0.2, mutation rate of 0.1, and 1,000 maximum iterations, for all subsequent GP analyses.

### Evaluating accuracy with varying models in the ensemble

**Fig. 3.**
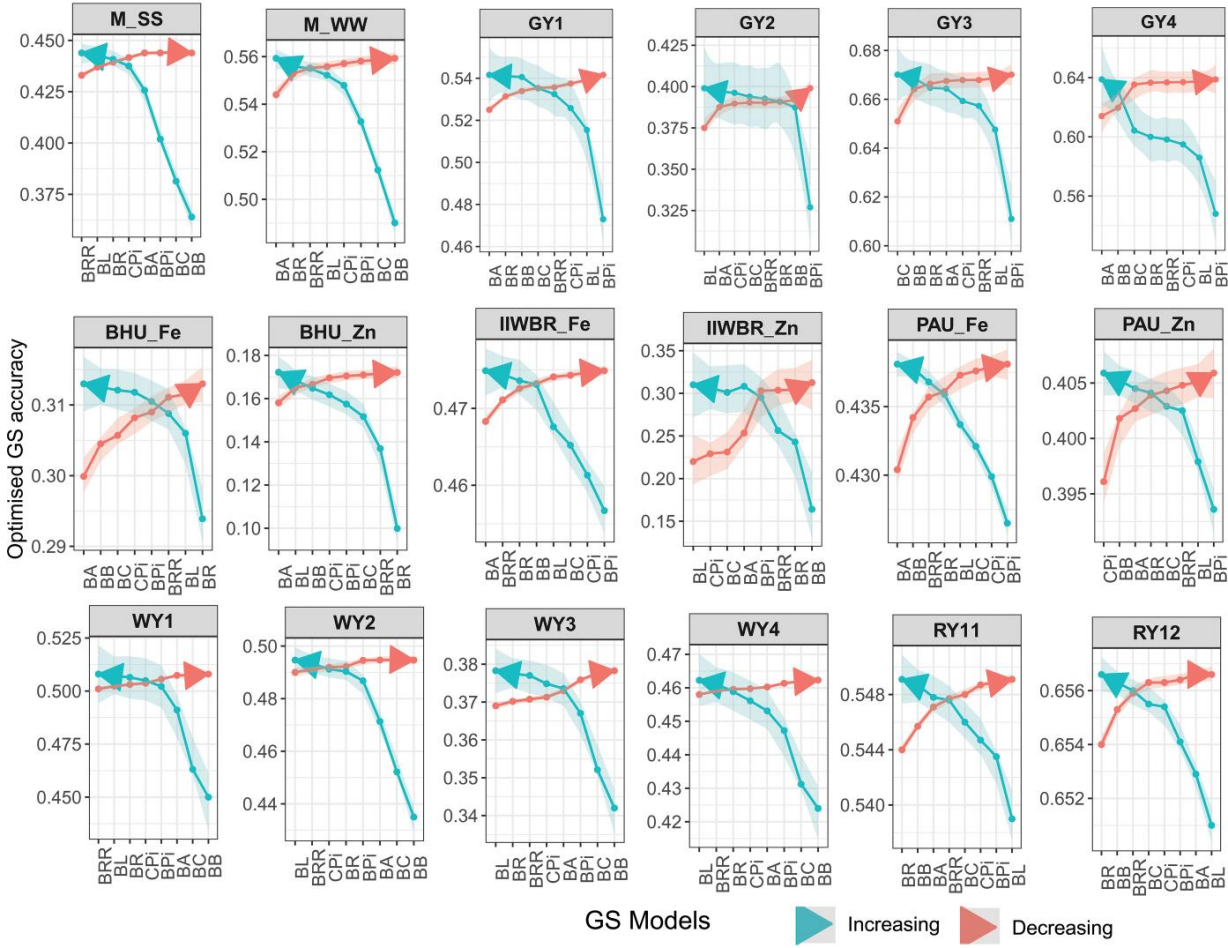
Trend in the genomic prediction accuracy of the ensemble model while individual Bayesian models are included one-by-one in decreasing order of accuracy and increasing order of accuracy in the ensemble framework. The objective function $f_{1}(w)$ is used to measure the genomic prediction accuracy in terms of PCC. The legend “Decreasing” represents that the models are included in decreasing order of accuracy and “Increasing” denotes that the models are included in increasing order of accuracy.

In order to determine whether the number of prediction models influences the prediction accuracy, the accuracy of the ensemble model (PCC obtained with objective function $f_{1}(w)$) was computed by taking 2, 3, 4, 5, 6, 7, and 8 models in a sequential manner. In addition, the models were included 1 by 1 in increasing order of accuracy as well as decreasing order of accuracy. It was observed that the rate of increase in accuracy of the ensemble model was higher while the models were included in increasing order of accuracy, whereas a lower rate of increasing in accuracy was found if the models were added in decreasing order of accuracy (Fig. 3). It was also noticed that the number of less accurate models required in the ensemble framework is always higher than the number of more accurate models to achieve same level of optimized accuracy. For instance, ensemble of BayesA, BayesR, and BayesRR resulted in PCC of 0.552 for M_WW. On the other hand, ensemble of BayesB, BayesC, BayesBpi, BayesCpi, and BayesL was required to achieve the same level of accuracy. Similarly in case of M_SS, ensemble of first 3 higher achieving models yielded PCC of 0.439 and to achieve the same accuracy, 5 lower predictive models such as BayesB, BayesC, BayesBpi, BayesA, and BayesCpi were required (Fig. 3). In case of BHU_Zn trait, ensemble of BayesA, BayesL, and BayesB achieved the same accuracy as achieved by ensemble of 5 less accurate models (BayesR, BayesRR, BayesC, BayesBpi, and BayesCpi) (Fig. 3). Therefore, it is better to use less number of more accurate models than more number of less accurate models in the ensemble to achieve a certain level of GP accuracy.

### Analysis of weight vs prediction accuracy

**Fig. 4.**
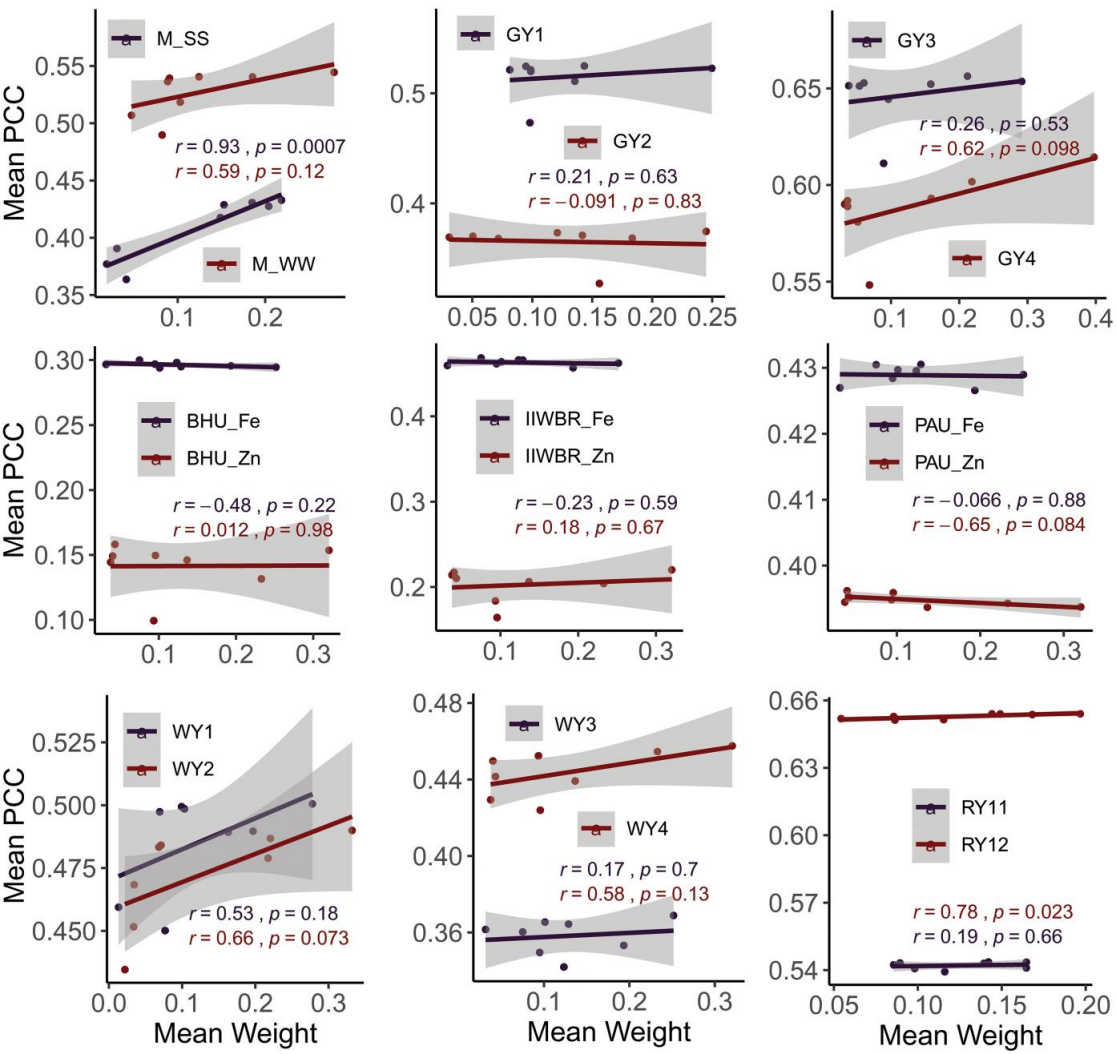
Correlation between PCC of the Bayesian models and the weights determined using the objective function $f_{1}(w)$.

In order to analyze whether higher weights were assigned to the model having higher GP accuracy, correlations between the weight and PCC (obtained with objective function $f_{1}$(*w*)) were analyzed over 100 experiments for the 8 Bayesian GP models. Out of 18 traits, significant association was observed (at 5% level of significance) only for 3 traits viz., M_WW (*r* = 0.93, *P* = 0.00074), IIWBR_Fe (*r* = 0.74, *P* = 0.037), and RY12 (*r* = 0.78, *P* = 0.023) (Fig. 4). Besides, negative association (although not significant) was also observed for some of the traits like GY2 (*r* = −0.091), GY4 (*r* = −0.22), and BHU_Fe (*r* = −0.39) (Fig. 4). Therefore, it may be said that the weight distribution of the GA optimization technique is dependent on the objective function that is to be minimized or maximized rather than the predictive ability of the individual model taken in the ensemble framework.

### Trade-off analysis between PCC and MSE

**Fig. 5.**
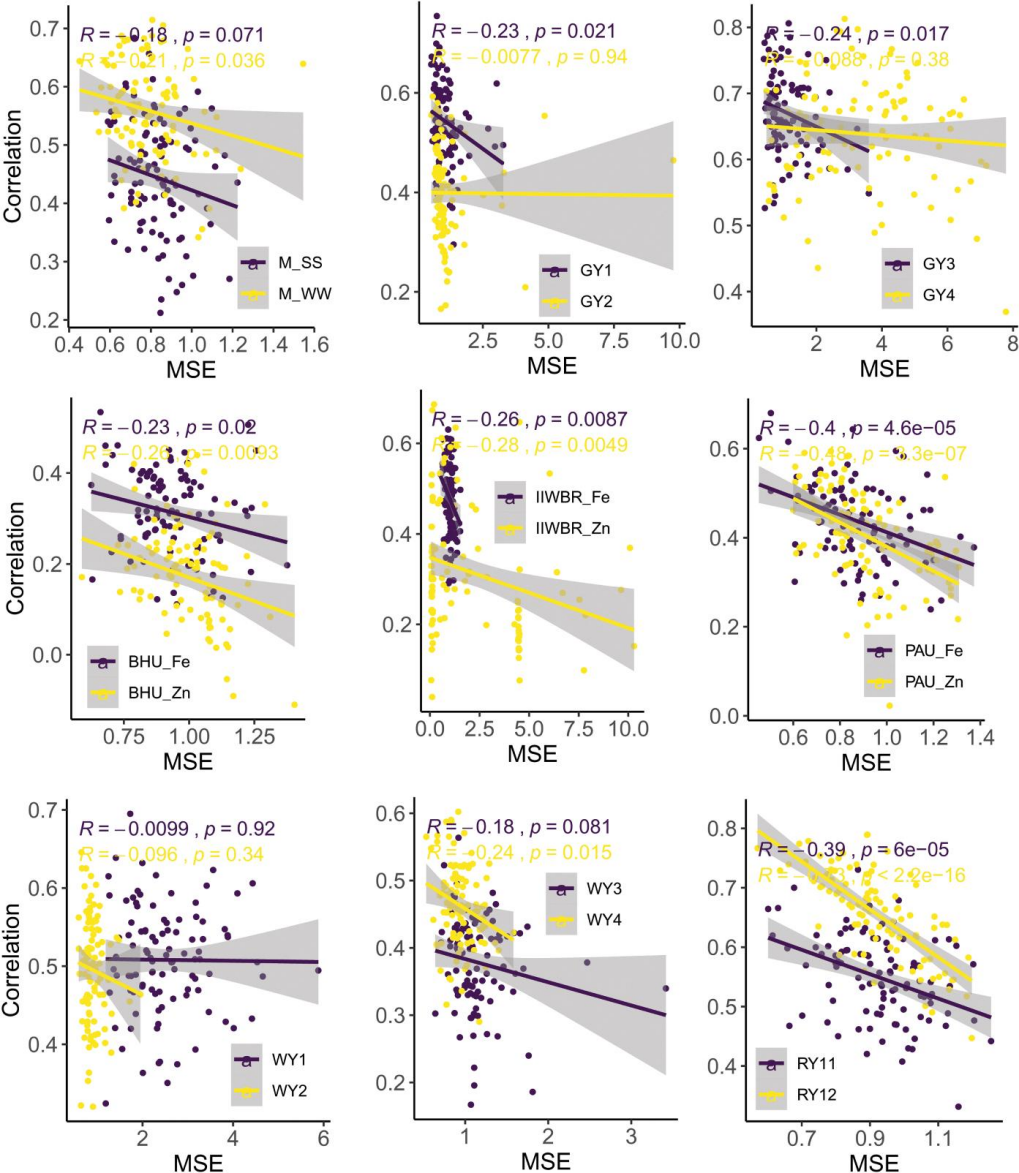
Correlation plots between PCC (y-axis) and MSE (x-axis) obtained using the objective functions $f_{1}(w)$ and $f_{2}(w)$ in the genetic algorithm, respectively. The 100 points in the figure represent genomic prediction accuracies of the EnBayes model across 100 repetitions of the experiment, where genetic algorithm was executed with 1,000 iterations.

**Fig. 6.**
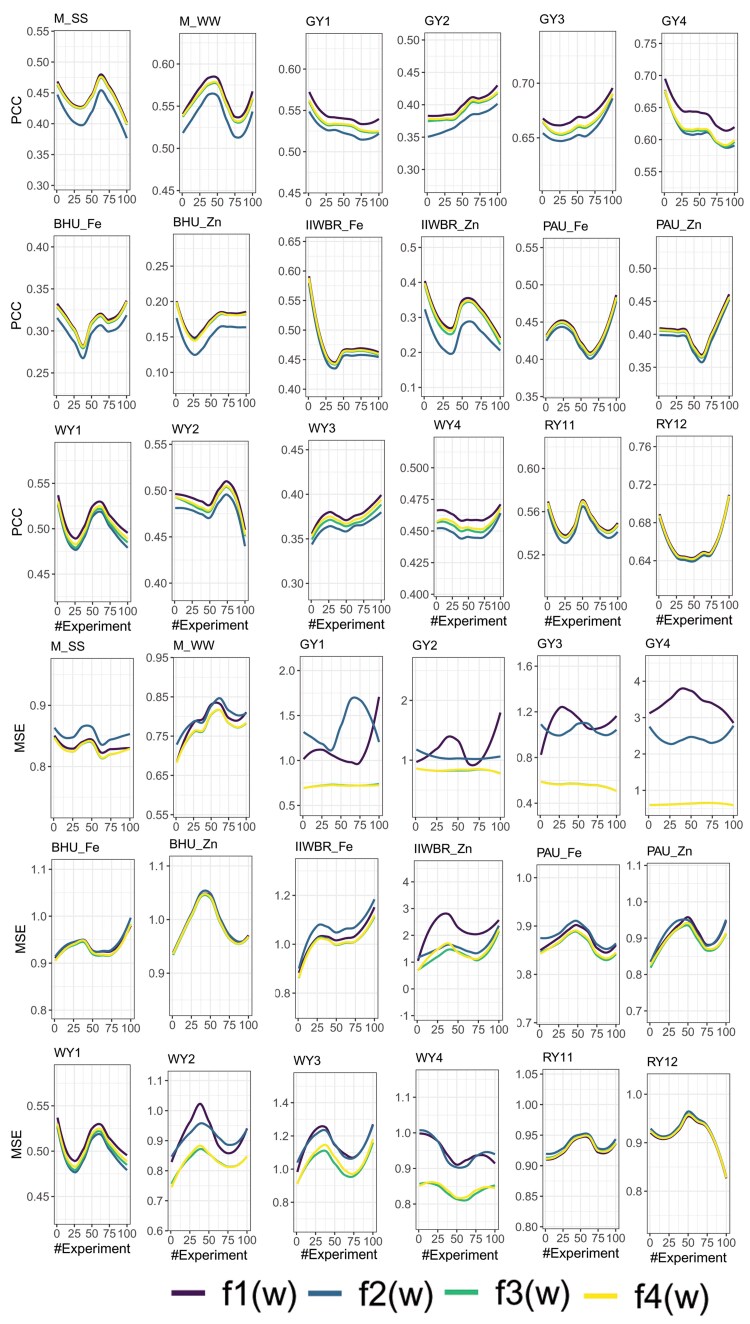
Trend in accuracy of genomic prediction of the ensemble model while the weights are assigned on the basis of 4 objective functions such as $f_{1}(w)$, $f_{2}(w)$, $f_{3}(w)$, and $f_{4}(w)$.

Both PCC and MSE have been utilized in existing studies to measure the GP accuracy. In order to find whether higher value of PCC produces less MSE and vice versa, correlation between the PCC (obtained with fitness function $f_{1}(w)$) and MSE (obtained with fitness function $f_{2}\;(w)$) of the ensemble model was analyzed. Negative correlation was found between MSE and PCC for all the 18 traits. However, no significant negative association was observed for 6 traits at 5% level of significance and for 11 traits at 1% level of significance (Fig. 5). Thus, it may be said that higher PCC may not always ensure lower MSE. While the weight assigned by GA on the basis $f_{1}(w)$ and $f_{2}\;(w)$ objective functions was used to find the PCC, it was observed for all the 18 traits that the PCC computed using the weights of $f_{2}\;(w)$ was always less than that obtained using the weight of $f_{1}\;(w)$ (Fig. 6). Similarly, when the weight assigned by the objective function $f_{1}\;(w)$ was used to find the MSE, it was observed that for most of the traits, the MSE was higher or at par with that of MSE obtained using the weights assigned by the objective function $f_{2}\;(w)$ (Fig. 6). Therefore, in order to make a balance between PCC and MSE, we considered the objective functions $f_{3}\;(w)$ and $f_{4}\;(w)$ which involves the maximization of PCC and minimization of MSE. It was observed that for both $f_{3}\;(w)$ and $f_{4}\;(w)$, there was a slight decline in the PCC (Fig. 6) but at the same time, the MSE was declined to a large extent (Fig. 6). More specifically, the PCC was declined by 1.6% and 1.86% while weight of $f_{3}\;(w)$ and $f_{4}\;(w)$ was respectively used, whereas the respective MSE was seen to be declined by 34.62% and 34.37% for the trait GY1 (Table 2). Similarly for the GY2 trait, though PCC was seen to be declined by 1.25% and 2.05%, the MSE was found to be declined by 28.97% and 29.33%, respectively (Table 2). Nonetheless, it was observed that the percentage of decline in MSE was significantly higher than that of PCC, barring exceptions (Table 2).

**Table 2.** Genomic prediction accuracy in terms of Pearson's correlation coefficient (PCC) and mean square error (MSE) for fitness functions $f_{1}\;(w)$, $f_{2}\;(w)$, $f_{3}\;(w)$, and $f_{4}\;(w)$.

	PCC				MSE			
Trait	$f_{1}\;(w)$	$f_{2}\;(w)$	$f_{3}\;(w)$	$f_{4}\;(w)$	$f_{1}\;(w)$	$f_{2}\;(w)$	$f_{3}\;(w)$	$f_{4}\;(w)$
M_SS	0.444	0.436	0.441	0.439	0.833	0.828	0.828	0.827
M_WW	0.559	0.543	0.554	0.552	0.789	0.764	0.771	0.770
GY1	0.541	0.530	0.533	0.531	1.098	0.721	0.718	0.721
GY2	0.399	0.388	0.393	0.391	1.174	0.830	0.834	0.829
GY3	0.670	0.661	0.663	0.662	1.118	0.561	0.558	0.559
GY4	0.638	0.610	0.614	0.612	3.423	0.627	0.625	0.626
BHU_Fe	0.313	0.306	0.311	0.309	0.935	0.929	0.932	0.930
BHU_Zn	0.172	0.162	0.169	0.169	0.989	0.987	0.989	0.988
IIWBR_Fe	0.475	0.467	0.472	0.471	1.026	1.008	1.009	1.009
IIWBR_Zn	0.312	0.261	0.305	0.298	2.259	1.054	1.347	1.234
PAU_Fe	0.438	0.432	0.434	0.435	0.869	0.854	0.858	0.856
PAU_Zn	0.406	0.398	0.403	0.402	0.904	0.886	0.893	0.889
WY1	0.508	0.496	0.502	0.498	2.529	2.199	2.116	2.195
WY2	0.495	0.484	0.489	0.488	0.915	0.824	0.831	0.829
WY3	0.378	0.365	0.374	0.369	1.149	1.016	1.047	1.027
WY4	0.462	0.451	0.456	0.454	0.949	0.835	0.843	0.838
RY11	0.549	0.546	0.548	0.547	0.926	0.933	0.928	0.929
RY12	0.656	0.654	0.655	0.655	0.917	0.921	0.919	0.919

### Analysis of bias in prediction accuracy

**Fig. 7.**
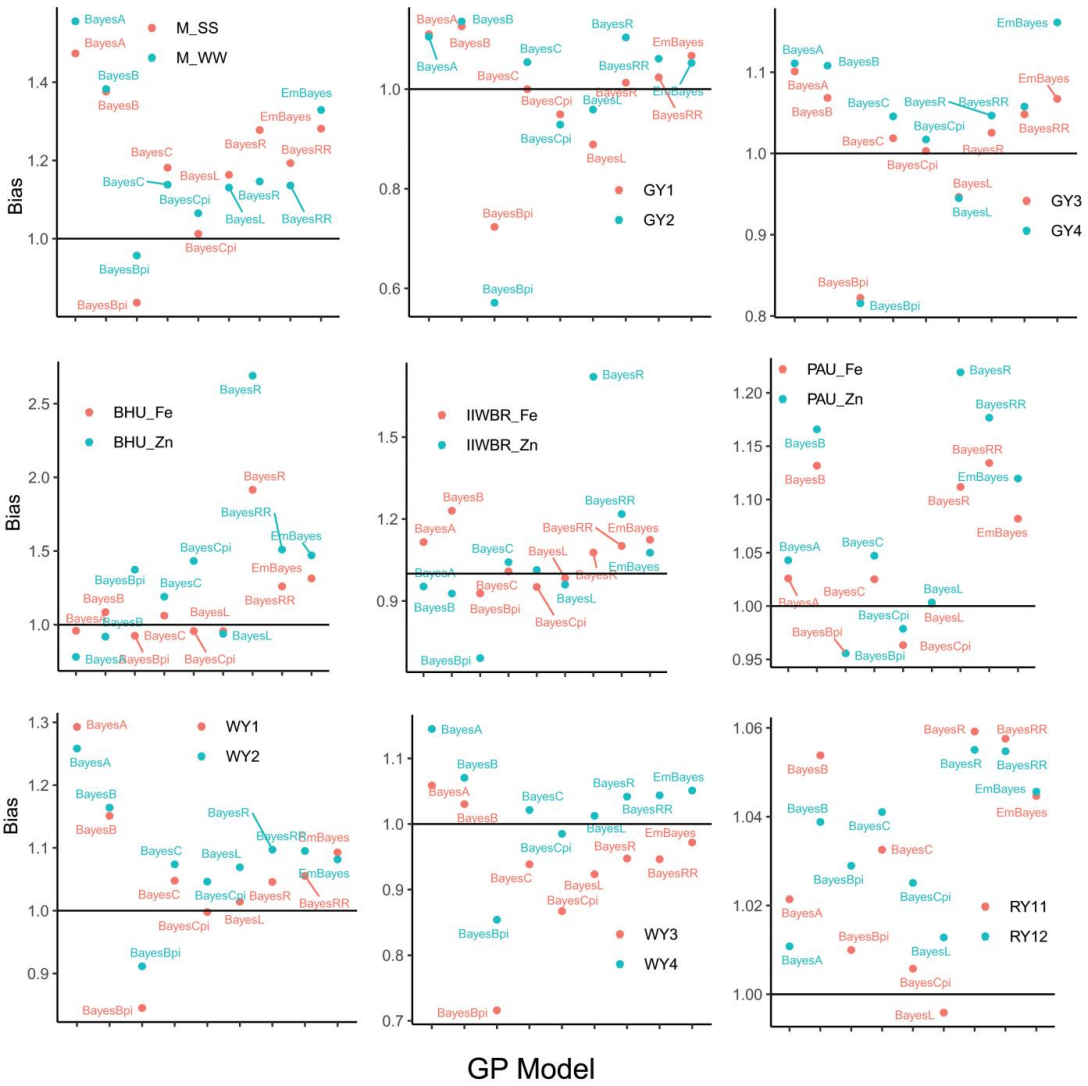
Estimation of bias in the genomic prediction accuracy for different Bayesian models and the ensemble model EnBayes.

To estimate the bias in GP accuracy, the observed phenotypic value was regressed upon the predicted phenotypic value and the coefficient of regression was estimated. It was observed that the GP accuracy of BayesA, BayesB, BayesC, BayesR, and BayesRR is mostly over-biased and the prediction accuracy of BayesBpi, BayesCpi, and BayesL is under-biased (Fig. 7). The GP accuracy of EnBayes (PCC obtained with objective function $f_{1}(w)$) was found to be over-biased (Fig. 7) which may be due to the fact that out of 8 models, 5 are over-biased which play a role in determining the bias of the ensemble model. Among the Bayesian models, BayesCpi was observed to be the least biased GP model (Fig. 7). Nonetheless, the bias in GP accuracy was found to be varied for models with different traits.

### Comparative analysis with other GP models

**Fig. 8.**
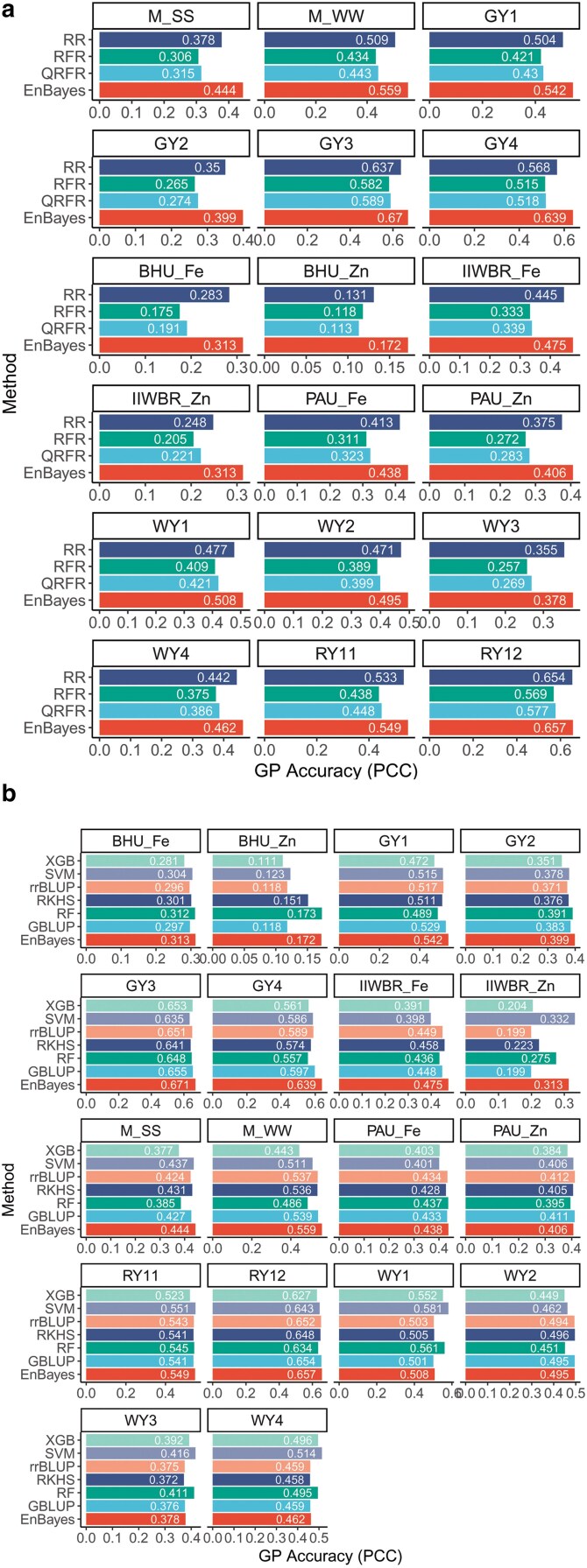
a) Comparison of genomic prediction accuracy obtained using meta-learning approach and the proposed ensemble model EnBayes. b) Comparison of genomic prediction accuracy of EnBayes with traditional (rrBLUP, GBLUP, RKHS) and ML genomic prediction models (SVM, RF, XGB).

Among the meta-learners, RR was found to achieve the higher GP accuracy as compared to QRFR and RFR (Fig. 8a). Nonetheless, the EnBayes model achieved the highest GP accuracy (PCC obtained with objective function $f_{1}(w)$) as compared to the meta-learners. In particular, the EnBayes secured 2–7% higher accuracy than that of best performing meta-learner RR model (Fig. 8a). In addition, for most of the datasets, the EnBayes outperformed other traditional and ML models as far as GP accuracy is concerned. For the M_SS and M_WW traits, EnBayes achieved PCC values of 0.444 and 0.559, respectively, surpassing the next-best methods (Fig. 8b). In GY1, EnBayes scored 0.542, outperforming GBLUP (0.529) and rrBLUP (0.517). Similarly, in GY2, EnBayes achieved a PCC of 0.399, exceeding RF (0.391) and GBLUP (0.383). For GY3 and GY4, EnBayes produced the highest PCC values of 0.671 and 0.657, respectively, followed by GBLUP (0.655, 0.643) (Fig. 8b). In the WY1, WY3, and WY4 traits, ML models outperformed classical statistical methods, with SVM achieving the highest PCC values of 0.581, 0.416, and 0.514, respectively (Fig. 8b). Notably, in WY2, the performance of RKHS (0.496), GBLUP (0.495), and EnBayes (0.495) was comparable. For the micro-nutritional traits of wheat, EnBayes showed higher accuracy for BHU_Fe (0.313), IIWBR_Fe (0.475), and PAU_Fe (0.438). Conversely, the highest PCC values for BHU_Zn (0.173), IIWBR_Zn (0.332), and PAU_Zn (0.412) were achieved by RF, SVM, and rrBLUP, respectively (Fig. 8b). For the rice yield traits, RY11 and RY12, SVM and EnBayes achieved the highest PCC values of 0.551 and 0.657, respectively (Fig. 8b). In summary, out of the 18 datasets, EnBayes achieved the highest GP accuracy in 10 datasets, followed by SVM in 5 datasets, and RF, RKHS, and rrBLUP in 1 dataset each.

### Analysis of the ensemble of Bayesian, BLUP, and ML models

**Fig. 9.**
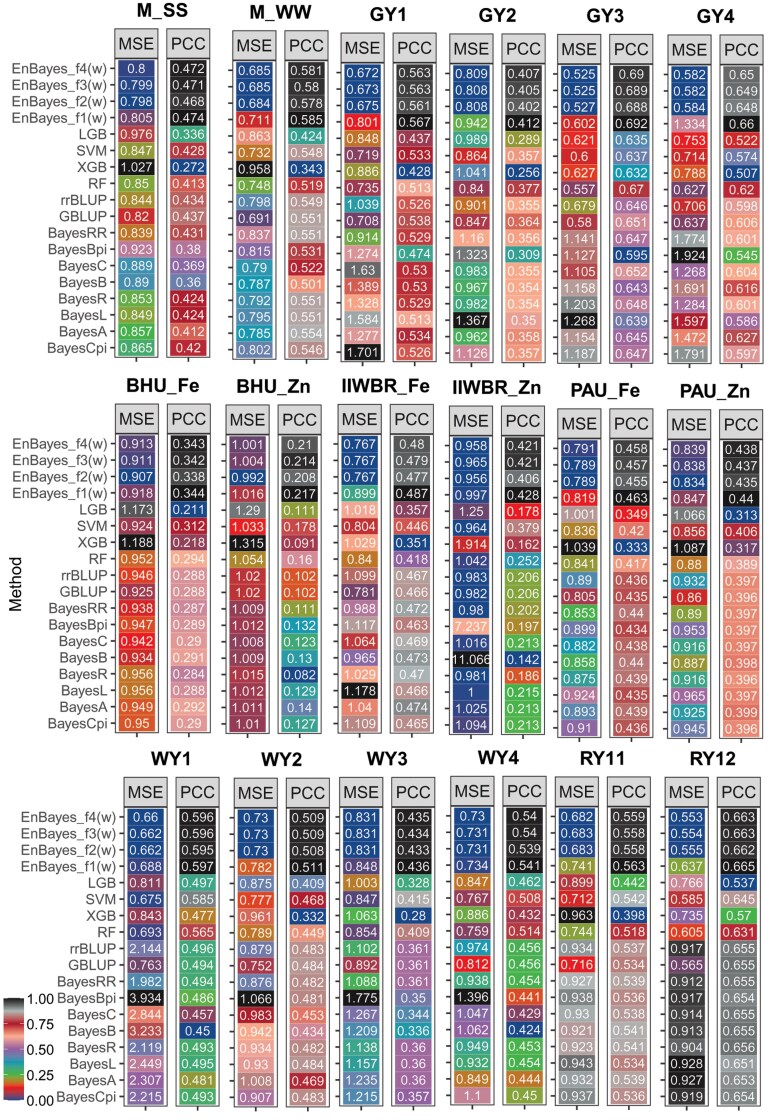
Genomic prediction accuracy (in terms of both PCC and MSE) of the proposed ensemble approach by considering 8 Bayesian models, 2 traditional models (rrBLUP, XGBLUP), and 4 machine learning models (SVM, RF, XGB, and LGB) in the ensemble framework.

From Fig. 8b, it was observed that SVM achieved higher GP accuracy in 5 traits, and RF and rrBLUP in 1 trait each. It is noteworthy that none of these models were included in the ensemble framework for GP prediction. Therefore, to further evaluate the performance of the proposed ensemble approach, rrBLUP, GBLUP, SVM, RF, XGB, and LGB models were included in the ensemble framework, in addition to the 8 Bayesian models. GP accuracies were evaluated using both PCC and MSE, considering all 4 objective functions $f_{1}(w)$, $f_{2}(w)$, $f_{3}(w)$, and $f_{4}(w)$ in the GA. The GP accuracies for all traits, models, and objective functions are shown in Fig. 9. Compared to the ensemble composed solely of Bayesian models, the Bayes + BLUP + ML ensemble achieved consistently higher GP accuracy across all 4 objective functions (Figs. 9 and 2). Specifically, under the objective function $f_{1}$(*w*), the Bayes + BLUP + ML ensemble achieved PCC values of 0.474, 0.585, 0.567, 0.412, 0.692, 0.660, 0.344, 0.217, 0.487, 0.429, 0.463, 0.440, 0.597, 0.511, 0.436, 0.541, 0.563, and 0.665 for the traits M_SS, M_WW, GY1, GY2, GY3, GY4, BHU_Fe, BHU_Zn, PAU_Fe, PAU_Zn, IIWBR_Fe, IIWBR_Zn, WY1, WY2, WY3, WY4, RY11, and RY12, respectively (Fig. 9). In contrast, the ensemble of only Bayesian models achieved lower PCC values of 0.444, 0.559, 0.542, 0.399, 0.670, 0.639, 0.313, 0.172, 0.475, 0.312, 0.438, 0.406, 0.508, 0.495, 0.378, 0.462, 0.549, and 0.657 for the respective traits (Fig. 2a). Similar improvements were also observed across other objective functions. Furthermore, the MSE obtained under objective functions $f_{1}(w)$, $f_{2}(w)$, $f_{3}(w)$, and $f_{4}(w)$ was consistently lower than that of the 8 Bayesian, 2 BLUP, and 4 ML models (Fig. 9). However, the MSE of $f_{1}(w)$ was higher among the 4 objective functions, as this objective function focused solely on maximizing the PCC, without considering MSE minimization (Fig. 9). This observation highlights that maximizing PCC alone does not guarantee MSE minimization. Conversely, the PCC obtained using $f_{2}(w)$ was lower compared to the other objective functions, as $f_{2}(w)$ involves only MSE minimization (Fig. 9). This further demonstrates that minimizing MSE does not necessarily ensure the maximization of PCC. Among the individual models, GBLUP achieved the highest GP accuracy for 3 traits (M_SS, GY3, WY2), BayesA for 7 traits (M_WW, GY1, GY2, GY4, IIWBR_Fe, WY1, RY11), RF for 2 traits (WY3, WY4), and BayesR and BayesRR for 1 trait each (RY12 and PAU_Fe, respectively), in terms of both PCC and MSE. The higher GP accuracy of the Bayes + BLUP + ML ensemble may be attributed to the diversity in predicted phenotypic values across models, which contributes unique variance and reduces redundancy. In contrast, the predictions from Bayesian models alone are highly correlated, which might have introduced redundancy in the ensemble, resulting in low GP accuracy.

## Discussion

We proposed an ensemble approach for GP, integrating 8 different Bayesian models utilizing GA optimization algorithm for weight assignment. This ensemble model demonstrated higher prediction accuracy across a variety of traits compared to individual Bayesian models. This may be due to the fact that ensemble learning captures a broader range of genetic effects by combining the strengths of multiple models, each tailored to different genetic architectures. Unlike individual models that may fail to capture the entire genetic variance, the ensemble method can account for a wider array of genetic effects, making it more robust for traits with complex genetic architectures.

Our study revealed that there is no significant linear relationship (barring exceptions) between the weights assigned by optimization techniques and the predictive accuracy of individual models. This is expected, as the GA does not optimize based on individual model performance, but rather on the overall ensemble prediction accuracy. In the ensemble framework, weights are assigned to the predicted phenotypic values generated by each model, and not to their individual accuracies. Therefore, a model with moderate individual accuracy may still capture unique predictive variance of the data, when combined with others and increases the overall ensemble accuracy. Conversely, models with high individual accuracy may provide predictions that are largely redundant with other models, reducing their contributions to ensemble performance and therefore receive lower weights. Furthermore, in the EnBayes model, the GP accuracy (maximization of PCC or minimization of MSE) was utilized as the objective function, where the combination of weights that yield higher accuracy is selected more frequently for reproduction, and over successive generations, the GA concentrates its search around the most promising regions of the solution space which leads to improvement in GP accuracy. Additionally, through crossover and mutation, the GA explores new combinations of model weights that might not be intuitive, where the crossover mixes good solutions to generate even better ones and mutation prevents the algorithm from getting stuck in local optima by introducing variation.

It was also found that ensemble of less number of more accurate models achieved higher GP accuracy than higher number of less accurate models. The GP accuracy was also found to be improved at a decreasing rate with increase in the number of individual models in the ensemble framework. It is, therefore, recommended that more accurate models be included first in the ensemble framework which may provide the optimized accuracy without even including less accurate models. Nonetheless, the full model, which combines 8 Bayesian models, demonstrates the highest prediction accuracy in most cases. With model selection, one must manually compare the performance of each individual model for every trait, which can be time-consuming. In contrast, EnBayes combines the models by assigning weights using a GA, eliminating the need to select a single best model for each trait. Although the computational cost is similar, as both approaches require evaluating all individual models, EnBayes reduces the human effort and decision-making complexity. Moreover, it produces higher prediction accuracy across all traits.

The GP accuracy has been measured either using the PCC between observed phenotypic trait and the predicted value or using the MSE of the test dataset in a cross-validation setup. However, none of the existing studies have evaluated the trade-off between these 2 metrics. In the present study, we initially employed 2 objective functions $f_{1}(w)$ and $f_{2}(w)$ to optimize the GP accuracy. It was found that the MSE and PCC are not found to be significantly negatively correlated (barring few exception), which implies that maximization of PCC measure does not ensure that MSE is minimized. Thus, in order to maximize the correlation and minimize the MSE simultaneously, we employed 2 more objective functions, i.e. $f_{3}(w)$ and $f_{4}(w)$. Although marginal reduction in correlation measure was observed for both $f_{3}(w)$ and $f_{4}(w)$, a substantial reduction in MSE was observed. Thus, if both the metrics are to be accounted for measuring GP accuracy, it is recommended that the objective function $f_{3}(w)$ and $f_{4}(w)$ to be used in an ensemble learning framework.

While assessing the bias in GP accuracy, it was observed that 5 out of 8 Bayesian models are over-biased which may be one of the probable reasons that the optimized GP accuracy was over-biased. Another plausible reason may be that all the Bayesian models are highly correlated. Therefore, it may be suggested that the models to be included in the ensemble framework should be varied in nature which has also been suggested by Gu et al. (2024).

Ensemble strategies have been previously explored in genomic prediction. For example, Ma et al. (2018) combined only 2 models such as deep convolutional neural networks and rrBLUP into an ensemble, where the model weights were optimized using PSO (Shami et al. 2022). However, PSO is known to be prone to getting trapped in local optima (Liu et al. 2005), potentially limiting the ensemble's predictive performance. In contrast, our approach integrates a more diverse set of models, including Bayesian methods, BLUP-based models, and ML algorithms, within the ensemble framework. We determined the optimal model weights using GA, which provides a more robust global optimization strategy and enhances the ensemble's predictive accuracy. In addition to weight optimization technique, different meta-learning approach has also been adopted for improving GP accuracy. For instance, Liang et al. (2021) proposed a stacking ensemble learning framework considering support vector regression, kernel ridge regression, and elastic net as base models, with linear regression serving as the meta-learner. The accuracy of the meta-learner outperformed the individual base learners. Similarly, Yu et al. (2021) proposed a GP model based on ensemble learning, which combined the predictions of 8 different ML regression models using a standard linear regression stacking approach. This model also demonstrated higher accuracy in GP compared to the individual models. In a recent study by Nascimento et al. (2024), a stacking ensemble learning model was used to enhance the prediction accuracy of key traits in *Coffea arabica* and observed higher accuracy for the meta-learner as compared to all the base learners. In this study, we compared the performance of both weight optimization strategy and meta-learning approach for GP and observed higher accuracy for the weight optimization strategy approach as compared to meta-learning approach. In fact, the meta-learning approach in this work showed less accuracy than many of the base learner which contradicts the existing studies (Liang et al. 2021; Yu et al. 2021; Nascimento et al. 2024). This may be due to the fact that base learners in the existing study are less correlated as compared to the present study where the Bayesian alphabets are highly correlated. In addition to the meta-learning approach, the proposed ensemble model was also compared with traditional GP models as well as ML algorithms. For most of the datasets, the ensemble approach achieved higher GP accuracy despite the exclusion of these models in the ensemble framework. On the other hand, the ensemble model was observed achieving higher GP accuracy while the traditional GP models (GBLUP, rrBLUP) and ML models (SVM, RF, XGB, LGB) were included in the ensemble framework.

## Summary

In this study, we utilized a weight optimization strategy to ensemble individual GP models for achieving higher GP accuracy. The GA was used to determine the weights assigned to individual models, where 8 Bayesian models were considered in the ensemble framework. Higher GP accuracy was observed for the developed ensemble model as compared to the individual prediction model. New objective functions were proposed to obtain better accuracy in terms of both PCC and MSE. It was inferred that few individual models with higher GP accuracy are better than that of more number of less accurate models. It was also observed that while the base models are highly correlated, then it is better to employ a weight optimization strategy rather than adopting meta-learning approach in the ensemble to get higher GP accuracy. The bias in GP accuracy of the ensemble model was observed to be determined by the base models considered in the ensemble framework. Nonetheless, the proposed ensemble approach is expected to supplement the existing efforts for improving the GP accuracy.

## Supplementary Material

jkaf150_Supplementary_Data

## Data Availability

All the processed phenotypic and genotypic datasets used in the study and source code are available at https://github.com/PrabinaMeher/EnBayes. Supplemental material available at G3 online.
