# Supplementary material for: Ensemble of Bayesian alphabets via constraint weight optimization strategy improves genomic prediction accuracy
Source: G3 (Bethesda). 2025 Jul 29;15(9):jkaf150. doi: 10.1093/g3journal/jkaf150 (PMC12405891; doi:10.1093/g3journal/jkaf150)

**Supplementary Figure S1**. Genomic prediction accuracy (PCC obtained with$f_{1}(w)$) for different combinations of hyper parameters (population size=200, 300, 500; mutation=0.05, 0.1, 0.15; crossover=0.2, 0.4, 0.8, iteration=1000, 5000) in genetic algorithm


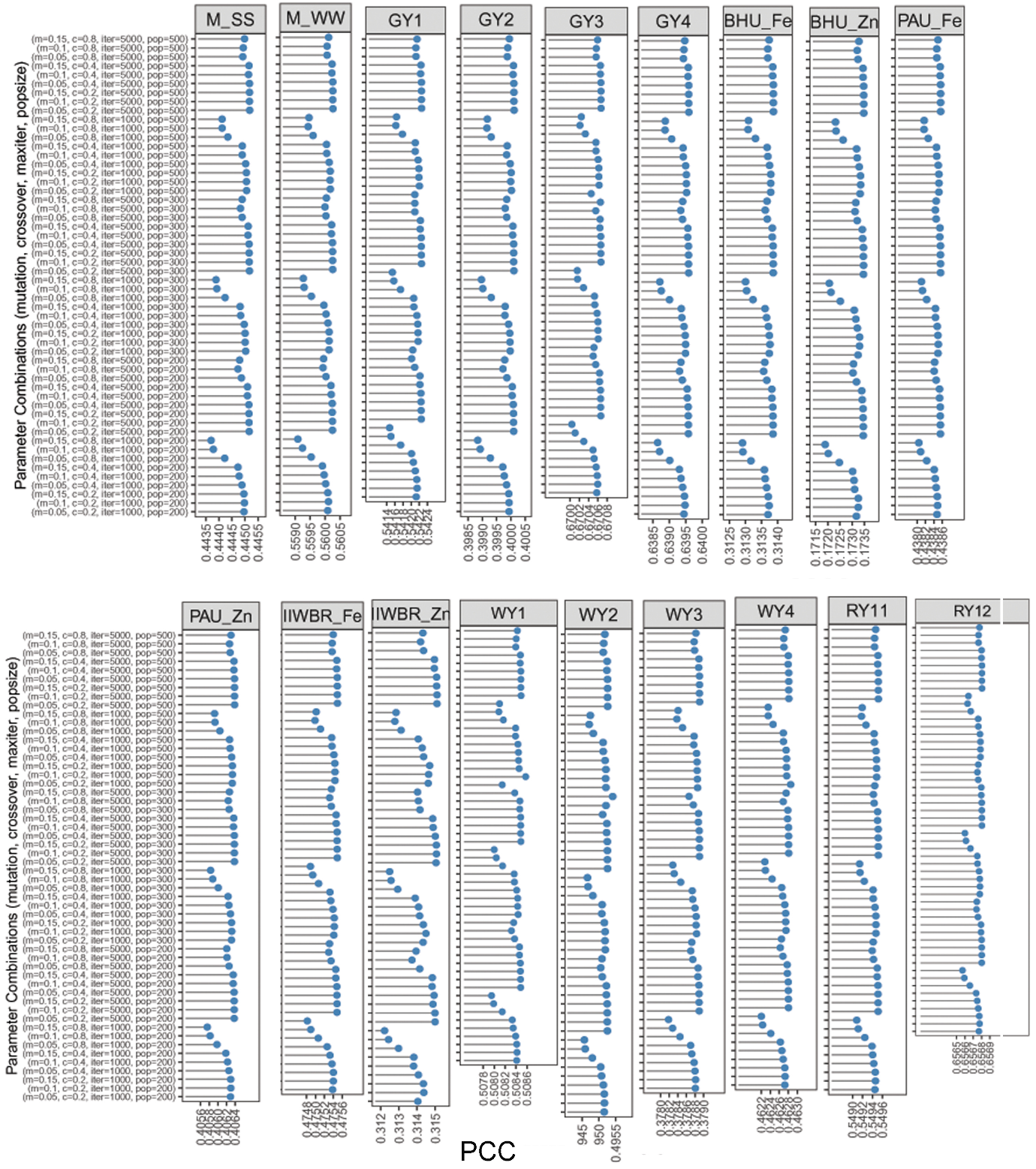

Supplement: jkaf150_Supplementary_Data [file jkaf150_supplementary_data.docx]
